# Arthroscopic fixation of ACL avulsion fracture in the saint pault hospital: A review of treatment outcomes: Cohort study

**DOI:** 10.1016/j.amsu.2019.07.008

**Published:** 2019-07-08

**Authors:** Tran Trung Dung, Hoang Gia Du, Nguyen Hoang Long, Le Manh Son, Dao Xuan Thanh, Dinh Ngoc Son, Nguyen Trung Tuyen, Do Van Minh, Nguyen Huy Phương, Vu Tu Nam, Pham Trung Hieu, Ma Ngoc Thanh

**Affiliations:** a Hanoi Medical University, Viet Nam; b Saint Paul University Hospital, USA; c Bachmai University Hospital, Viet Nam; d Vietduc University Hospital, Viet Nam; e Hanoi Medical University Hospital, Viet Nam

**Keywords:** Arthroscopic fixation, Anterior cruciate ligament avulsion fracture, Suture fixation, Fiber wire technique

## Abstract

**Background:**

The purpose of this research is to evaluate the results of arthroscopic suture fixation with fiber wires used as treatment for ACL avulsion fracture, and to determine how effective such a technique is when it comes to restoring of knee function.

**Materials and methods:**

This prospective study involves 28 patients, who underwent arthroscopic fixation of displaced ACL avulsion fractures at Saint Paul Hospital (Hanoi) from January 2014 to March 2018.

**Results:**

The first three weeks were not marked with any abnormalities associated with post-operative sutures and hematomas, infectious complications were not detected either. Post-operative displacement of fracture fragments did not take place among the patients involved in the study. At the 3-month follow-up, the average IKDC score was 90.7 (range: 76–100), and the average Lysholm score was 93.6 (range 82–100). The percentage of excellent scores was 42.9% (12 patients), good scores accounted for 50% (14 patients), while fair/poor scores accounted for 3.6% each (1 patient on each score). The percentage of excellent/good scores was 92.9% in total.

**Conclusion:**

This study shows that ACL avulsion fracture can be treated effectively by arthroscopic suture fixation with fiber wires. In fact, this technique may restore knee function and stability.

## Highlights

• Evaluating the results of arthoscopic suture fixation with fiber wires used as treatment for ACL avulsion fracture.
• The study involves 28 patients.
• The ACL avulsion fracture can be treated effectively by arthoscopic suture fixation with fiber wires. It may restore knee function and stabiity.

## 1 Introduction

The anterior cruciate ligament (ACL) injury accounts for 33–92% of injuries in the capsular-ligamentous apparatus [1]. The ACL injury is diagnosed subsequently to injuries accompanied by hemarthrosis in 10–65% of cases [2]. Displaced ACL avulsion fractures are not a common pathology. In adult patients, ACL injury accounts for 1–5% of all injuries [3], and is usually the result of road traffic accidents, sports, and less often, household and occupational activities. Late diagnosis and improper treatment of acute injury may spark rapid degenerative processes in the joint, and cause a significant work decrement in patients [4]. These fractures are currently classified by the Meyers and McKeever classification system [5], according to which type I fractures are nondisplaced; type II fractures produce displacement of the anterior margin while the posterior part is still seated onto the tibia; and type III fractures are completely displaced. This classification system was improved by Zaricznyj, who added type IV with complete fragment comminution [6,7].Conservative treatment is indicated for patients with type I fractures. In other cases, when patients have type III and type IV fractures or when other concomitant injuries are present, a stable internal fixation is indicated [8]. Progress in arthroscopy brought up more possibilities for arthroscopic fixation in patients with type II fractures, so the number of such services increased [8,9]. To this end, there is no priority method for fixing ACL. A 100% reliable fixation device is also a thing that does not exist. Fixing methods described in the literature imply the use of screws, wires, and anchors. They are known for encouraging results [7,8,[10], [11], [12], [13], [14], [15]], but they are not without certain drawbacks. Currently, arthroscopic suture fixation with fiber wires is the most common technique [[16], [17], [18], [19], [20], [21]]. However, there are concerns about potential postoperative complications.

The purpose of this research is to evaluate the results of arthroscopic suture fixation with fiber wires used as treatment for ACL avulsion fracture, and to determine how effective such a technique is when it comes to restoring of knee function.

## 2 Materials and Methods

This prospective study involves 28 patients, who underwent arthroscopic fixation of displaced ACL avulsion fractures at Saint Paul Hospital (Hanoi) from January 2014 to March 2018. Injury distribution among the patients by age and sex is presented in Table 1.

**Table 1:** Injury Distribution Among Patients by Age and Sex, n = 28.

Age (years)	Sex				Total	
	Male		Female			
	No.	Percent	No.	Percent	n	Percent
<20	1	12.5	1	5.0	2	7.1
20–39	4	50.0	18	90.0	22	78.6
40–59	3	37.5	1	5.0	4	14.3
Total	8	100.0	20	100.0	28	100.0

The majority of 24 patients, or 85.7%, were injured in road traffic accidents. When this study started, 75.0% of patients had their injuries present for less than 10 days, and only 10.7% of them (3 patients) had injuries for 20 days. The ACL fracture diagnosis implied the consideration of patient complaints, history taking, and physical examination. A clinical examination of patients was carried out using the Anterior Drawer Tests, Lachman Tests, Pivot Shift Tests, Valgus and Varus Stress Tests. Patients underwent radiography and computed tomography (CT) of the knee joint for verification purposes, as well as for being later classified by Meyer's and McKeever's criteria. Thus, 17 patients were classified to have type III fractures, 3 patients were classified to have type II fractures, and 6 patients were classified to have type IV fractures. In the intraoperative period, diagnostic arthroscopy was applied to assess associated injuries and complications, as well as to determine the extent of ACL injury. The isolated ACL avulsion fracture was a concomitant in 22 patients, 5 patients had an ACL rupture, and only one patient had medial meniscus tear. Clinical and pathological characteristics of patients are presented in Table 2.

**Table 2:** Clinical and pathological characteristics of patients.

Parameter		No. cases	Percent
Activity at Injury	Traffic accident	24	85.7%
	Work accident	1	3.6%
	Daily activities accident	2	7.1%
	Sports injuries	1	3.6%
Timing of Surgery	≤10 days	21	75.0%
	11–20 days	4	14.3%
	>20 days	3	10.7%
Involved Knee	Right side	13	46.4%
	Left side	15	53.6%
Meyers and McKeever classification of ACL alvusion fracture	Type II	3	10.7%
	Type III	17	60.7%
	Type IV	8	28.6%
Concomitant injuries	Isolated ACL alvusion fracture	22	78.6%
	ACL Rupture	5	17.9%
	Medial Meniscus tear	1	3.6%

The arthroscopic suture fixation with fiber wires was performed on each patient involved in the study. Surgery was performed under spinal anaesthesia with the following instruments on the tray: the Stryker Arthroscopy Instrument Set, an ACL tibial tunnel, and the Arthrex fiber wire suture.

The patient was placed supine with the knee flexed 90°. The thigh was secured using a tourniquet placed around it. An anterolateral portal was established for the introduction of arthroscope. The hematoma was evacuated until reasonable visualization was possible. Once the pathology was visualized, an anteromedial portal was established using needle localization. An arthroscopic probe was then used to dislodge any clotted blood or debris at the site of fracture. A synovial resector was used to debride the region further and to remove any debris from the fracture bed. Once the fracture site was debrided, the probe was used to attempt a reduction. Once the fracture was reduced, a pin was placed percutaneously from a medial parapatellar position to hold the fracture reduced. After reducing the fracture and holding it in position with the Steinmann pin, the ACL tibial tunnel was used to advance a guide pin through the anteromedial tibial metaphysic to the joint on the medial side of the fracture fragment. A second guide pin was advanced starting 1–2 cm lateral to the first hole on the tibial cortex, so that it entered the knee on the lateral side of the fracture fragment. The fiber wire was passed througt the tibial tunnel, and a knot was secured tightly to the tibial cortex. All wounds were sutured in layers tightly. The knee joint has been drained for 24–28 h using an active aspirator.

Patients were placed in a hinged brace locked at 0° flexion for the first 4 weeks. They were allowed to perform passive or active-assisted range of motion exercises when prone with knee flexed to 90°. Patients could bear weight as tolerated with the brace locked at 0°. Crutches were generally discontinued on tenth postoperative day. At the end of the fourth week, the brace was removed to start the closed-chain quadriceps exercises. At week 8, easy straight-ahead running was initiated, but pivot-twist maneuvers were avoided until at least the 12th post-operative week.

The first three weeks were devoted to the assessment of the wound, hematoma, infection, radiograph images. Time period between third week and the third month was the period when the study implied the assessment of the range of motion (ROM), stability, and radiographic images. At the 3-month follow-up, the focus was laid on ROM, clinical testing (Anterior Drawer Tests, Lachman Tests, Pivot Shift Tests, Valgus and Varus Stress Test), KT-1000 testing, radiography, International Knee Documentation Committee (IKDC) scores, and the Lysholm Knee score.

Study written according to The STROCSS Statement: Strengthening the Reporting of Cohort Studies in Surgery [22].

## 3 Results and discussion

The first three weeks were not marked with any abnormalities associated with post-operative sutures and hematomas, infectious complications were not detected either. The radiographs displayed no cases of post-operative displacement of fracture fragments caused by thread rupture.

At the 3-month follow-up, the average IKDC score was 90.7 (range: 76–100), and the average Lysholm score was 93.6 (range 82–100). Twelve patients, or 42.9%, reported normal knee movement, 15 of patients, or 53.6%, reported nearly normal, and only one patient, or 3.6%, did not notice any progress (Table 3). Thus, surveys showed that arthroscopic suture fixation througt the tibial tunnel with fiber wires gives a high subjective estimate of physical condition and physical activity level among patients (see Table 4).

**Table 3:** Post-operative Knee Range of Motion, Ligament Examination, KT-1000 Scores and IKDC-Final, data taken after 3 post-operative months.

Parameter		A (Excellent)	B (Good)	C (Fair)	D (Poor)
Range of Motion (ROM)	Flexion	16 (57.1%)	10 (35.7%)	1 (3.6%)	1 (3.6%)
	Extension	28 (100%)	–	–	–
Clinical Testing	Anterior Drawer	22 (78.6%)	6 (21.4%)	–	–
	Lysholm Score	23 (82.1%)	5 (17.9%)	–	–
	Pivot Shift	25 (89.3%)	3 (10.7%)	–	–
	Valgus Stress	28 (100%)	–	–	–
	Varus Stress	28 (100%)	–	–	–
KT-1000 Score		23 (82.1%)	5 (17.9%)	–	–
IKDC-Final		12 (42.9%)	14 (50%)	1 (3.6%)	1 (3.6%)

**Table 4:** Relationship between IKDC-Final, age of patients, timing of surgery, onset stage and concomitant injuries.

Parameter		A (Excellent)	B (Good)	C (Fair)	D (Poor)	No.
Age	<20	1	1	–	–	2
	20–39	10	10	1	1	22
	40–59	1	3	–	–	4
Timing of surgery	≤10 days	9	10	1	1	21
	11–20 days	2	2	–	–	4
	>20 days	1	2	–	–	3
Type by Meyers and McKeever Classification	Type II	3	–	–	–	3
	Type III	7	9	1	–	17
	Type IV	2	5	–	1	8
Concomitant injuries	Isolated ACL alvusion fracture	11	10	1	–	22
	ACL Rupture	1	3	–	1	5
	Medial Meniscus tear	–	1	–	–	1

The 2000 IKDC protocol was used to perform fair evaluation. This protocol allows evaluating the knee function by using a combination of clinical and radiological tests. Thus, patients were divided into four groups: A group, B group, C group, and D group. Patients showing excellent outcomes were placed in group A, those with good outcomes were placed in group B, with fair outcomes were place in group C, and patients with poor outcomes were placed in group D. By analogue, patients were grouped according to KT-1000 scores. Table 3 shows that only one patient had fair/poor flexion, or 3.6% in each group. The rest of the cases accounted for excellent and good results (excellent results dominated over the good ones). The same picture is true for KT-1000 scores (group A – 82.1%, group B – 17.9%). By the IKDC-final, 12 patients, or 42.9%, had excellent results, 14 patients, or 50%, had good results, and only one patient had fair/poor results, or 3.6% in each group. Thus, the percentage of excellent/good scores was 92.9% in total.

Table 1 shows the relationship between the IKDC-final and such parameters as the age of patients, timing of surgery, onset stage, and concomitant injuries. The uneven distribution of patients by these indicators between groups precludes a more complete analysis. However, note that patients with fair/poor flexion were 20–39 years of age, operated in less than 10 days after the injury took place, and had type III (group C by IKDC-final) and type IV fractures (group D). The first patient had isolated ACL avulsion fracture as a concomitant injury, while the second patient had an ACL rupture to play this role. Thus, patient with the worst IKDC-final had a more serious injury from the very start.

When it comes to patients with type III and type IV fractures, and often with type II fractures, rapid surgical interventions are the only thing that guarantees full recovery of knee functions. Currently, preference is given to arthroscopic treatment that allows reducing postoperative injuries and rehabilitation period. The literature gives many different methods of ACL fixation, including those that imply the use of cancellous screws, staples, sutures, fiber wires and bio-absorbable suture anchors [23]. All methods have certain drawbacks, for example, cancellous screws and staples cannot be used for comminuted fractures [24,25]. The use of fabric wire is associated with the risk of fracture displacement due to thread rupture during the postoperative period [26]. The arthroscopic suture fixation has been recently the most common method with various improvements being developed [16]. Thus, there is a whole range of works reporting excellent and good results with high functional scores obtained using suture fixation [17,27]. Ahn and Yoo report a Lysholm score of 95.6 [18], Song et al. indicate a 89.5 score [19], while Jadhav and Gotecha report a high of 98.6 [21]. Seon et al. compared the Lysholm score obtained by ACL fixation with cancellous screws with that obtained by suture fixation (91.7 vs 92.7) [20]. Boutsiadis et al. reported the average IKDC score of 91.1 (range: 77 to 100) and the average Lysholm score of 95.4 (range: 83 to 100) [16].

In those studies devoted to arthroscopic suture fixation through tibial tunnel with fiber wires, subjective knee scores (Lysholm sore: 93.6; IKDC score: 90.7) were similar to those given in the literature. Objective IKDC 2000 scores and KT-1000 scores obtained by measurement are comparable with the subjective knee scores reported by the patients. Thus, arthroscopic suture fixation with fiber wires applied to acute injury gives 92.9% of excellent and good outcomes.

## 4 Conclusions

This study did not confirm the concerns expressed in the literature about possible postoperative complications during arthroscopic suture fixation of ACL avulsion fracture. None of the patients had them. Despite the higher complexity of surgery with arthroscopic fixation, minimal invasiveness and shorter rehabilitation period that distinguish this method from open fixation make it preferable for the treatment of ACL avulsion fracture. Suture fixation can be indicated for type II, type III and type IV fractures, as well as for comminuted fractures when screws and braces cannot be used.

The IKDC 2000 scores were excellent in 12 and good in 14 patients, who underwent arthroscopic suture fixation of ACL avulsion fracture at the Hanoi Saint Paul Hospital. This accounted for 92.9% of all operated patients. The worst result was demonstrated by a patient with type IV fractures and concomitant ACL rupture. The average subjective IKDC and Lysholm scores were high, indicating physical activity regarded as good.

This study shows that ACL avulsion fracture can be treated effectively by arthroscopic suture fixation with fiber wires. In fact, this technique may restore knee function and stability. Thus, it can be recommended for acute injuries.

## Compliance with ethical standards

Every research study involving human subjects must be registered in a publicly accessible database before recruitment of the first subject.

## Conflicts of interest

The authors declare that they have no conflict of interest.

## Funding

There is no funding source.

## Ethical approval

This article does not contain any studies with human participants or animals performed by any of the authors.

## Provenance and peer review

Not Commissioned, internally reviewed.

## Ethical approval

It was waived at our institution.

## Sources of funding

Authors declare that they have no third party findings.

## Author contribution

Tran Trung DUNG study concept or design, data collection, data analysis and interpretation, writing the paper.

Hoang Gia DU study concept or design, data collection, data analysis and interpretation, writing the paper.

Nguyen Hoang LONG study concept or design, data collection, data analysis and interpretation, writing the paper.

Le Manh SON study concept or design, data collection, data analysis and interpretation, writing the paper.

Dao Xuan THANH study concept or design, data collection, data analysis and interpretation, writing the paper.

Dinh Ngoc SON study concept or design, data collection, data analysis and interpretation, writing the paper.

Nguyen Trung TUYEN study concept or design, data collection, data analysis and interpretation, writing the paper.

Do Van MINH study concept or design, data collection, data analysis and interpretation, writing the paper.

Nguyen Huy PHƯƠNG study concept or design, data collection, data analysis and interpretation, writing the paper.

Vu Tu NAM study concept or design, data collection, data analysis and interpretation, writing the paper.

## Conflicts of interest

Authors declare that they have no conflict of interests or other financial interests.

## Trial registry number

D: 125051.

https://www.crd.york.ac.uk/prospero/#myprospero.

## Guarantor

Tran Trung DUNG (Hanoi Medical University, trantrung.dung@yandex.ru).

## Appendix A Supplementary data

AMS-D-19-00028_DataProfile AMS-D-19-00028_DataProfile

The STROCSS Guideline The STROCSS Guideline
